# Significance of Endogenous Antimicrobial Peptides on the Health of Food Animals

**DOI:** 10.3389/fvets.2021.585266

**Published:** 2021-06-28

**Authors:** Yewande O. Fasina, Temitayo Obanla, George Dosu, Sierra Muzquiz

**Affiliations:** Department of Animal Sciences, North Carolina Agricultural and Technical State University, Greensboro, NC, United States

**Keywords:** antimicrobial peptide, food animals, host immune system, antimicrobial resistance, gut health

## Abstract

Acquired resistance to in-feed antibiotic growth promoters continues to be an imperative problem in the livestock industries, thereby necessitating continuous pursuit for alternatives. Antimicrobial peptides (AMPs) represent a critical part of the host's innate immune system and have been documented to have immunomodulatory activity. Increasing research evidence suggests that in contrast to antibiotics, AMPs exert broad-spectrum antibacterial activity in a manner that reduces bacterial acquisition of resistance genes. This review summarizes current knowledge on the protective effects of endogenous (natural) AMPs in the gastrointestinal tract of food animals. Factors limiting the efficacy of these AMPs were also discussed and mitigating strategies were proposed.

## Introduction

Antimicrobial peptides (AMPs), also referred to as host (endogenous) defense peptide (HDPs), are tiny cationic peptides that occur naturally in a variety of plants, animals, and microbes (1). Typically, AMPs have a broad-spectrum activity against microorganisms, and have the ability to kill multidrug-resistant bacteria (2). They also exert immunomodulatory activities such as recruiting and activating cells of the innate and adaptive immune system (3). The molecular and cellular mechanisms underlying the activity of AMPs involve inducing changes in membrane-associated targets (such as cell wall biosynthesis and cell division) or on cytoplasmic targets such as macromolecular synthesis in cells (4). In food animals such as pigs and poultry, the antimicrobial and immunomodulatory activities of AMPs synergistically culminate in beneficial physiological effects such as improvements in growth performance, nutrient digestibility, and intestinal morphology, in addition to a diversified healthy gut microbiota (2).

Scientists have classified AMPs into five major families based on their structural compositions and amino sequence: defensins, cathelicidins, hepcidins, histone-derived peptides, and the fish specific piscidins (5, 6). Several reports show that AMPs from fish exhibit similar antimicrobial and immunomodulatory properties to those found in other organisms (7).

Until recent, conventional antibiotics were included in feed for food animals as prophylactics to prevent and control foodborne and disease-causing pathogens, and to promote growth (8). However, the ability of bacteria to be intrinsically resistant to certain antibiotics and/or acquire resistance to antibiotics *via* mutations in chromosomal genes and by horizontal gene transfer has dampened the effectiveness of antibiotics (9). The consequent evolution of antibiotic-resistant bacterial strains and their transmission to humans, has regrettably threatened food safety and public health (10–13). Therefore, governmental legislation(s) have been enacted to phase out (or halt) the incorporation of antibiotics (and other antimicrobial drugs) in animal feed (14). The use of antimicrobials in food animal production worldwide is geographically heterogeneous due to the existence of different regulations governing their use (15). For instance, incorporation of antibiotics into animal feed has been banned in the European Union and USA since 2006 and 2017 respectively, while countries such as China, Vietnam, Brazil, and Bangladesh have only limited their use (15–17). In spite of these efforts, it has been projected that worldwide antimicrobial consumption by animals will rise by 67% between 2010 [from 63,151 (±1,560) tons] and 2030 [to 105,596 (±3,605) tons] to sustain animal health (18). Presently, the consequential rise of antibiotic-resistant bacteria annually results in the death of about 700,000 people worldwide, and this catastrophe has been projected will be killing about 10 million people yearly by 2050, on a global basis (19). Accordingly, continuous effort is being made to identify alternative non-antibiotic effective strategies for controlling enteric pathogens in food animals.

Studies have shown that AMPs could control and prevent infectious diseases, particularly against antibiotic-resistant bacteria (20, 21). This review therefore explores the role(s) of various classes of AMPs in maintaining the health of food animals (such as pigs, cattle, fish, and poultry), with more emphasis on gut health.

## Endogenous Antimicrobial Peptides That Enhance Porcine Health

### Cathelicidins

Cathelicidins exhibit both pro-and anti-inflammatory activity through complex interactions that modulate various immune processes such as apoptosis, inflammasome activation, and phagocytosis (22). Proline-Arginine-39 (PR-39) is a small cationic cathelicidin that is found in the pig's intestinal cells, bone marrow, lymphoid tissues (e.g., thymus and spleen), and leukocytes (23). PR-39 has broad-spectrum antimicrobial activity against enteric pathogenic bacteria such as *Enterococcus faecalis, E. coli*, and *Bacillus subtilis* (24), and may play a role in mitigating intestinal inflammation and diarrhea in pigs (25). Enterocolitis in piglets is a diarrheal disease caused by multiple bacteria including *Escherichia coli* and *Salmonella* spp. The disease has endangered the sustainability of the pork sector through production losses such as reduced feed efficiency, increased mortality, treatment costs, and lack of confidence by consumers on the safety of the meat (pork) products. Enterocolitis results in intestinal inflammation that is often characterized by massive infiltration of neutrophils, followed by septicemia and death (23). Cathelicidins probably mitigate enterocolitis by reducing neutrophil adhesion and rolling by ***blocking the recruitment of neutrophils*** (26). ***This*** ***effect is accomplished through the inhibition of ubiquitin proteasome-mediated*** ***I***_***k***_***B*α** ***degradation on endothelial cells, which in turn downregulates the expression of ICAM-1*** ***and VCAM-1*** (26). ***Thus, PR-39 could alleviate excessive inflammation during intestinal*** ***diseases by limiting the influx of neutrophils, and may partially replace current expensive*** ***therapies***such as fluid therapy, antibiotic and anti-inflammatory therapy. Furthermore, PR-39 may re-establish epithelial integrity and intestinal barrier function by promoting intestinal wound healing and angiogenesis.

### β-Defensins

Defensins are AMPs that are cysteine-rich with six conserved cysteine residues that form three pairs of disulfide bridges (6). In vertebrate animals, defensins are classified into three sub-groups, namely α-defensins, β-defensins, and θ-defensins, based on distribution of cysteine residue that forms the disulfide bridges and the length (27). Defensins produced by cells in the course of innate immune response serve as signals that initiate, mobilize, and amplify adaptive immune host defenses (28). β-defensins are known to play an essential role in innate and adaptive immunity due to their antimicrobial, chemotactic, and regulatory activities (28). β-***defensins*** ***exert inhibitory effects on pathogenic bacteria, fungi, mycobacteria, and enveloped viruses*,** ***particularly by creating pores on the microbial membrane surface to increase cellular*** ***permeability*** (29). They are also considered chemotactic for T-lymphocytes and immature dendritic cells (30).

The synthesis and secretion of AMPs is triggered by molecules such as cytokines, lipopolysaccharide, β-glucans, and bacterial DNA that signal the presence of potentially pathogenic microorganisms (31). Expression of β-defensins is prevalent in the tissues that control the immune system, such as the spleen, thymus, and lymph node (28). Certain nutrients such as isoleucine, arginine, glucose, Ca^2+^, and zinc are able to regulate the expression and synthesis of β-defensins (31–33). For instance, Mao et al. (31) supplemented pig diets with 0.5% L-arginine and observed a significant increase (*P* < 0.05) in the expression of porcine β-defensin-2 gene in the oral epithelium, tongue, ileum and inguinal lymph node, and that of porcine β-defensin-3 gene in the ileum and inguinal lymph node. Supplementing swine diets with amino acid and cation mixtures that can optimally enhance the expression and synthesis of intestinal β-defensins will likely improve gut health and resistance to pathogens, and consequently reduce (or eliminate) the use of antibiotics in swine diets.

### Cecropins

Cecropins are naturally occurring AMPs in the small intestine of pigs. This AMP possess bactericidal activity against both Gram-negative and Gram-positive bacteria, fungi, and viruses (34). Their physiological and pathophysiological relevance is inherent in their ability to modulate membrane permeability. ***The mechanisms of*** ***action involve creopins forming partially selective ion channels or binding to*** ***negatively charged membrane lipids to form a closely packed layer that renders*** ***membranes permeable*** (35). Weaning is a critical stage for piglets because it is associated with changes in the architecture and function of the gastrointestinal tract, as well as changes in enteric microbiota and immune responses (36). A common disease of piglets is post-weaning diarrhea which is characterized by watery feces and reduced performance, thereby causing economic loss to farmers (37). Wu et al. (35) demonstrated that cecropins improved the performance of piglets challenged with *E. coli* and increased the population of *lactobacilli* strains—a healthy bacteria population in the intestine. Furthermore, the study revealed that creopins increased the concentrations of serum immunoglobulins and inflammatory cytokines [such as interleukin (IL)-1 and IL-6] which are indicators of an activated immune system (35). The ability of Creopins to act as adjuvants that stimulate humoral and antigen-specific cytotoxic-T-cell responses positions them as an alternative to antibiotics in diets for weaned piglets.

## Endogenous Antimicrobial Peptides That Enhance Bovine Health

### Bovine Cathelicidins

Cattle are raised as livestock for meat, milk, and other multi-purpose uses. Infectious diseases, including respiratory, intestinal and reproductive maladies, lameness, and mastitis are major concern (38). Because of the ability of cathelicidins to recognize and kill invading pathogens and stimulate immune defenses (39), they are promising alternatives to antibiotics and drugs for the control of diseases in cattle. Bovine mastitis is a disease condition in which the udder of the cow is infected by a wide range of bacteria, including *S. aureus, E. coli, S. uberis*, non-aureus *Staphylococci, Klebsiella* sp., *Streptococcus dysgalactiae*, and *Mycoplasma bovis* (40). Production-related symptoms associated with mastitis include decreased milk quality and yield. The family of bovine myeloid antimicrobial peptides (BMAPs) BMAP−27, BMAP-28, and BMAP-34 are synthetic host defense peptides derived from naturally occurring bovine cathelicidins, and ***they have shown antibacterial activity against pathogens*** **such as** ***S. aureus***, ***B. megaterium***, ***E. coli***, ***P.aeruginosa***, and *S. enterica* serotype Typhimurium that cause mastitis (41). Increased levels of cathelicidins in milk were found in 75% of cows naturally infected with *S. uberis*, whereas cathelicidins were absent in healthy cows (42). Therefore, cathelicidins have been proposed as alternative diagnostic markers for mastitis compared to the expensive and sometimes inaccurate traditional bacteriological culture methods to detect somatic cell counts.

Another important cathelicidin in animal health is the synthetic Bac2A that is derived from bovine bactenecin, a 12-amino acid cyclic cationic antimicrobial peptide that contains one intramolecular disulfide bond, through the substitution of two cysteine residues for two alanine residues (43). Synthetic Bac2A exhibits antibacterial activity with minimum inhibitory concentrations (MICs) ranging between 2 and 32 μg/mL against Gram-negative bacteria, and between 0.25 and 16 μg/mL against Gram-positive bacteria (43). Innate defense regulator (IDR)-1018 is another synthetic cathelicidin derived from bovine cathelicidin bactenecin (44). At concentrations of 20 μg/mL, IDR-1018 decreased LPS-induced TNF-α pro–inflammatory response in monocytes by 89% (44). It also has considerable activity against *S. aureus* (MIC 5 μg/mL), although little activity against *P. aeruginosa* was reported (44). Such innate immune regulatory characteristics make IDR-1018 an ideal alternative to traditional in-feed prophylactic antibiotics.

### β-defensins

Bovine respiratory disease (BRD) also known as bacterial pneumonia disease, affects production in the beef industry with a significant economic loss (45). Predisposing factors for BRD in beef cattle include viral infections and the stresses of weaning, transportation, castration and inclement weather conditions (45). Under predisposing conditions, the innate immune system is compromised and bacterial composition in the nasal cavity is altered in a manner that increases in the population of pathogenic bacteria (46). Typically, cattle BRD is controlled through metaphylactic use of antibiotics, but this intervention does not consider the underlying roles of stress and viral infection in the disease (45). ***Tracheal antimicrobial peptide (TAP), a 38-amino acid cationic peptide, is a*** **β*-defensin*** ***produced by epithelial cells lining of the respiratory tract and other mucosal surfaces in cattle*** ***and protect the respiratory mucosal surfaces from infection*** (46). Research findings suggest that antibacterial activity of TAP during BRD is compromised by stress-induced elevations in cortisol concentration (46). Similarly, Vulikh et al. (45) reported that the bactericidal activity of β-defensin naturally produced in bovine airways during pneumonia is suppressed by glucocorticoid (stress) and viral infection. We propose that effective control of BRD will require the implementation of management strategies that reduce animal stress, and administration of potent endogenous-source AMPs.

## Endogenous Antimicrobial Peptides That Enhance Poultry Health

Most studies documenting the antimicrobial activity of AMPs in poultry species has been done with chickens. Therefore, the role of AMPs in chicken health is discussed in this section.

### Cathelicidins

Modulation of inflammatory response *via* the activation of a wide variety of TLRs during infections is regulated by cathelicidins (47). Three main cathelicidins, namely chCATH-1, −2 −3 (also called fowlicidin-1, −2, and −3), have been identified in chickens and confirmed to have antimicrobial activity against Gram-negative and Gram-positive bacteria, including antibiotic-resistant strains (48). The presence of cathelicidins in lymphoid tissues suggest a possible involvement of AMPs in the maturation and development of adaptive immunity (3). A study by Veldhuizen et al. (49) revealed that chicken cathelicidins have antibacterial activity against methicillin-resistant *Staphylococcus aureus* (MRSA). Specifically, Veldhuizen et al. observed about two log reduction in MSRA count when a concentration of 0.6 mM of cathelicidins was used, and found a complete eradication of the bacteria when the concentration of cathelicidins was increased to 2.5 mM. Goitsuka et al. (50) concluded that chCATH-B1 is an antimicrobial defense element whose cellular localization is pivotal to protection against invasion by viable microbes *via* the mucosal M cell gateway. Furthermore, Bommineni et al. (51) showed that CATH1 possess excellent immunomodulatory properties with a strong capacity to ***specifically chemo-attract neutrophils without affecting the migration of monocytes or*** ***lymphocytes***.

Most studies have shown that Avian AMPs are active against a broad-spectrum of bacteria. The AMPs have a strong ability to modulate the host response to infection and inflammation. However, inability of host organism to produce adequate quantity of AMPs (also known as host defense peptides, HDPs) could limit the functions of HDPs. Bioactive compounds such as butyrate and vitamin D_3_ could be used as dietary supplements to increase the production of AMPs. In chickens, butyrate has been proven to be a strong inducer of HDP expression *in vitro* and *in vivo*. A study conducted by Sunkara et al. (52), revealed that supplementing chicken diets with butyrate and a plant extract containing forskolin, which is an adenylyl cyclase agonist, showed a strong synergy in augmenting HDP expression in the crop and jejunum of chickens. We propose that supplementing the diets of poultry with bioactive volatile fatty acids such as butyrate and vitamin D3 could promote HDP synthesis, host immunity, and disease resistance.

### B-Defensins

Avian β-defensins (AvBD) are characterized based on their antimicrobial capability against a broad spectrum of pathogens including bacteria and fungi (53). In chickens, 14 AvBD genes have been identified (AvBD1 to AvBD14), and their expression has been confirmed in various tissues including the bone marrow, respiratory tract, skin, digestive tract, and reproductive organs, and in cells like heterophils (54, 55). β-defensin gallinacin-6 (Gal-6) has been reported to show *antimicrobial activity against food-borne pathogens* such as *Campylobacter jejuni, Salmonella enterica* serovar Typhimurium, *Clostridium perfringens*, and *E. coli* (54). There is also some evidence that the expression of some AvBDs is enhanced with oviductal growth during sexual maturation by the effects of estrogen (56). Accordingly, as hens age, the increasing expression of AvBDs as the oviduct grows may be important in protecting egg contents from pathogenic microorganisms.

### Ovodefensins and Gallin

Ovodefensins belongs to the family of β- defensins antimicrobial peptides containing conserved glycine and six cysteine residues (57). Ovodefensins are expressed in large amount in many parts of the chicken oviduct (57). The ovodefensins differ from classical vertebrate defensins in the spacing of amino acids within the six-cysteine sequence motif, and are slightly shorter in length having only 39–41 amino acids. Whenham et al. (56), indicated that ***ovodefensins*** ***caused a 98% reduction in Escherichia coli CFU/mL at 100 lM, and about 40 and*** ***90% reduction in the viability of avian pathogenic E.coli 078:H9 and S. aureus*,** ***respectively***.

Gallin, a peptide with a 41-residue protein is a composition of hen egg white that helps to protect the chicken embryo during its development in the egg (57). Gallin is synthesized in the magnum and shell gland of the oviduct, and is deposited into the egg albumen (58). Antibacterial assays confirmed that ***gallin at a concentration of 0.25*** **μ*M was*** ***active against E. coli***, but no additional antibacterial activity was observed against the other Gram-positive or Gram-negative bacteria tested (57, 58).

## Endogenous Antimicrobial Peptides That Enhance Fish Health

Antimicrobial peptides have been found to play immunomodulatory roles in fish species to provide defense against pathogenic attack (7). Natural AMPs such as piscidins, defensins, hepcidins and histone-derived peptides have been found in fish, thereby making fish a major source for AMPs (59).

### Piscidins

Piscidins are natural AMPs in the fish which are generally active against various microorganisms (mostly bacteria) and also possess anti-fungal, anti-viral and anti-parasitic activities (60, 61). The ***mode of action by piscidins is to inhibit further growth and*** ***development of pathogens by penetrating and destroying the spores*** (62). Other findings have indicated piscidins expression contributes to phagocytic activities. Thus, the intracellular release of piscidins by granulocytes aids in the gut health and defense against pathogenic attack (63). Furthermore, piscidins can mitigate pathogenic activities in extreme conditions due to the hemolytic activity conferred by the amphiphilic α-helical cationic structure (64). Research evidence has also suggested that the immunomodulatory effects of piscidins observed in fish are similar to those exhibited in mammalian species such as mice (65).

### Defensins and Cathelicidins

β-defensins remain the only type available in fish species (66). β-defensins are also active against some fish specific-viruses like the Singapore grouper iridovirus (SGIV) and the viral nervous necrosis virus [VNNV; (67)]. Research reports have indicated that β-defensins found in Atlantic cod can influence ***antimicrobial activity in phagocytes***, thereby providing multiple functions to the innate host defense (7, 68). Cathelicidin play significant role in immunity as an *anti-bacterial* host defense. Its expression has been observed during embryonic development where it provides some fish species with a first line of response against pathogenic bacteria such as *P. aeruginosa, V. anguillarum, E. coli*, and *Lactobacillus* spp. (69).

### Hepcidins

Hepcidins are normally expressed in the liver in the adult fish albeit its expression occurs early during development in some fish species (70). Fish have two types of hepcidins: hamp1 and hamp2 (7). Research reports suggest that the role of hamp1 focuses on iron metabolism regulation, whilst that of hamp2 is mostly antimicrobial in nature (71). Hypoxia in fish hinder hepcidin to actively fight against microorganism attack (72). On the other hand, hepcidin expression is induced by high levels of iron in both zebrafish and sea brass (73, 74). Hepcidin is believed to ***increase resistance to microbial infection by*** ***preventing the release of iron from macrophages, and preventing the absorption of iron in*** ***the small intestine***.

### Histone-Derived Peptides

Histone-derived peptides ***exhibit a variety of host defense mechanisms against broad*** ***spectrum of both Gram-positive and Gram-negative bacteria***, and have been shown to be secreted in fish species when epidermal damage occurs before (75). Some histone-derived peptides require binding with other antimicrobial peptides in order to express their maximum antimicrobial potential. For instance, histone-derived peptides containing NETs (neutrophil extracellular traps) exhibit strong potential to trap and kill bacteria in some fish species. However, very little information is known about the mode of action of histone-derived peptides against pathogenic attack and immunomodulatory responses in fish, compared to other antimicrobial peptides (7).

## Mitigating Microbial Resistance to Endogenous Antimicrobial Peptides in Food Animals

The antimicrobial activities of endogenous AMPs include inactivation of pathogenic microbes, reinforcing the antimicrobial barrier function of epithelial cells particularly in the gut, and linking innate immunity to adaptive immunity (76, 77). The cationic nature of endogenous AMPs facilitate their ability to exert antimicrobial effect(s) on pathogens. Although the precise mechanism(s) by which AMPs cause bacterial cell death is still poorly understood, The amphipathic interaction between the AMPs (net-positively charged) and the negatively charged microbial cell surfaces allow the insertion of AMPs into the microbe's cell membrane (78). In bacterial, the net-negative charge in the cell membrane is due to their constituent phospholipids (like cardiolipin, phosphatidylserine, and phosphatidyl glycerol), lipopolysaccharide, and lipotechoic acids (79). To cause cell death, AMPs bind directly to the lipopolysaccharides of Gram-negative bacteria and lipoteichoic acids of Gram-positives, and then depolarize the cell membranes to make them permeable (80, 81). Regardless, mounting research evidence show that pathogenic microbes can develop the ability to evade antimicrobial effects of AMPs. Microbes utilize various mechanisms to evade the antimicrobial activity of AMPs. For instance, bacterial resistance mechanisms to AMPs include (i) membrane modification through electrostatic repulsion of AMPs by alanylated teichoic acids, aminoacylated peptidoglycan, or amine compound-added lipid A, (ii) binding of AMP and inactivation by mechanisms such as surface shedding, (iii) active removal of AMPs from the bacterial cell by efflux pumps, (iv) proteolytic degradation of AMPs by extracellular proteases such as elastase and gelatinase, (v) upregulation of bacterial AMP resistance genes by global transcriptional regulators, and (vi) downregulation of AMP expression or upregulation of host AMP-degrading proteases such as cathepsins (81–83). However, compared to antibiotics, it is believed that endogenous AMPs contribute less to microbial resistance to drugs because of their stable structure, antimicrobial activity, selective toxicity, wide-spectrum and high-efficiency, and minimal side effects (84, 85).

Microbial resistance to endogenous AMPs can be mitigated through dietary modulation strategies that increase the expression of AMPs, enhance the stability of AMPs at epithelial surfaces, and upregulate redox proteins present at the epithelial surfaces. In a recent review, Wu et al. (85) summarized the nutrients that have been established to upregulate the expression of AMPs in the gut mucosa. These include (i) amino acids such as branched-chain amino acids, Arginine, and tryptophan that upregulate the expression of AMPs in the gut *via* the Sirt1–ERK1/2–90RSK (sirtuin-1-extracellular regulated protein kinase1/2- 90-kDa ribosomal S6 kinase), GPCR–MAPK (G protein-coupled receptor-mitogen-activated protein kinase), and NO signal or mTOR (nitric oxide signal- mammalian target of rapamycin) pathways; (ii) fatty acids such as short-chain fatty acids, medium-chain fatty acids, and long-chain fatty acids contribute to the expression of AMPs by directly influencing histone acetylation and the GPCR–MAPK signal pathway; (iii) lactose from plants, and polysaccharides from plants and bacteria, and (iv) trace elements and vitamins such as zinc, lactoferrin, cholecalciferol (vitamin D3) and its metabolite 1,25-dihydroxycholecalciferol [1,25(OH)2-D3], vitamin B-3, and vitamin A. Furthermore, the presence of thioredoxin, a redox protein present at the intestinal epithelial surface, is known to facilitate the antimicrobial activity of AMPs (86). A recent report by has also suggested that redox active AMPs can undergo reversible oxidation after interaction with some electron transport chain proteins and their products, or interaction with the periplasmic redox system of Gram-negative bacteria (77). Further research is required to identify molecular-based strategies that will exploit the reversible oxidation state of endogenous AMPs, for the synthesis of AMPs that will have improved stability in the gut mucosa and other epithelial membranes.

## Conclusion

Considerable research has been done to identify endogenous AMPs in food animals, and their mechanisms of antimicrobial activity (Table 1). The main advantages of using AMPs include their broad spectrum of activity, fast action against pathogenic bacteria, and probably decrease in bacterial acquisition of resistance genes contrary to the situation often seen with the use of conventional antibiotics. Different approaches for using AMPs to manage animal health include (i) combined dietary administration of antimicrobial peptides and conventional antibiotics, and (ii) dietary supplementation of their precursor nutrient molecules (such as amino acids, fatty acids, and some micronutrients) at optimum concentrations that can increase AMP expression in the animal's body. The possibility to manipulate the oxidative state of some AMPs to achieve increased stability at in the gut mucosa and other epithelial membranes require further investigation. It was concluded that AMPs can could at least partially replace conventional antibiotics in food animal production, thereby improving the quality and microbiological safety of animal meat and egg products intended for human consumption.

**Table 1:** Endogenous antimicrobial peptides in food animals.

**Species**	**Antimicrobial peptide**	**Mode of action**	**Endogenous source**
Porcine	Cathelicidin—Proline-Arginine-39 (PR-39)	Block the recruitment of neutrophils through the inhibition of ubiquitin proteasome-mediated IkBα degradation on endothelial cells	Intestinal cells, bone marrow, lymphoid tissues, and leukocytes
	β-Defensins	Creating pores on the microbial membrane surface to increase cellular permeability	Tissues and cells of the immune system
	Creopins	Modulate membrane permeability by forming partially selective ion channels, or binding to negatively charged membrane lipids to form a closely packed layer that renders membranes permeable	Small intestine
Bovine	Cathelicidins (synthetic endogenous-source)—Bovine myeloid antimicrobial peptides (BMAPs), Bac2A, and IDR-1018	Antibacterial activity against pathogens	Different tissues of the body and milk
	β-Defensin—Tracheal antimicrobial peptide (TAP)	Prevent infection at the respiratory mucosal surfaces through bactericidal activities	Epithelial cells lining the respiratory tract and other mucosal surfaces
Poultry	Cathelicidins, namely chCATH-1, −2 −3 (also called fowlicidin-1, −2 and −3),	Exert antibacterial activity by serving as a chemo-attractant to neutrophils, without affecting the migration of monocytes or lymphocytes	Lymphoid tissues in chickens
	β-Defensins—AvBD1 toAvBD14	Provides broad-spectrum antimicrobial activity	Expressed in various tissues, including the reproductive organs, bone marrow, respiratory tract, skin, digestive tract, and in cells like heterophils
	Ovodefensins	Antibacterial activity against *E. coli* and *S. aureus*	Expressed throughout the chicken oviduct
	Gallin	Antibacterial activity against *E. coli*	Found in egg albumen
Fish	Piscidins	Bacteriostatic—inhibit further growth and development of pathogens by penetrating and destroying the spores	Various tissues in the fish
	β-Defensins	Contributes to antimicrobial activity in phagocytes	Various tissues in the fish
	Cathelicidin	Antibacterial	Embryonic tissue in fish
	Hepcidins—Hemp1 and Hemp2	Increase resistance to microbial infection by preventing the release of iron from macrophages, and preventing the absorption of iron in the small intestine	Predominantly expressed in the liver of adult fish
	Histone-derived peptides	Broad-spectrum antibacterial activity	In Fish

## Author Contributions

All authors listed have made a substantial, direct and intellectual contribution to the work, and approved it for publication.

## Conflict of Interest

The authors declare that the research was conducted in the absence of any commercial or financial relationships that could be construed as a potential conflict of interest.

## References

[1] Konno Y Ashida T Inaba Y Ito T Tanabe H Maemoto A. Isoleucine, an essential amino acid, induces the expression of human β defensin 2 through the activation of the G-protein coupled receptor-ERK pathway in the intestinal epithelia. Food Nutr Sci. (2012) 3, 548–55. 10.4236/fns.2012.34077

[2] Wang G Li X Wang Z. APD3: the antimicrobial peptide database as a tool for research and education. Nucleic Acids Res. (2016) 44:D1087–93. 10.1093/nar/gkv1278 26602694 PMC4702905

[3] Achanta M Sunkara LT Dai G Bommineni YR Jiang W Zhang G. Tissue expression and developmental regulation of chicken cathelicidin antimicrobial peptides. J Anim Sci Biotechnol. (2012) 3:15. 10.1186/2049-1891-3-15 22958518 PMC3436658

[4] Haney EF Straus SK Hancock REW. Reassessing the host defense peptide landscape. Front Chem. (2019) 7:43. 10.3389/fchem.2019.00043 30778385 PMC6369191

[5] Wang G Li X Wang Z. APD2: the updated antimicrobial peptide database and its application in peptide design. Nucleic Acids Res. (2009) 37:933–7. 10.1093/nar/gkn823 18957441 PMC2686604

[6] Lei J Sun L Huang S Zhu C Li P He J. The antimicrobial peptides and their potential clinical applications. Am J Transl Res. (2019) 11:3919–31. 31396309 PMC6684887

[7] Masso-Silva JA Diamond G. Antimicrobial peptides from fish. Pharmaceuticals (Basel). (2014) 7:265–310. 10.3390/ph7030265 24594555 PMC3978493

[8] Li Z Hu Y Yang Y Lu Z Wang Y. Antimicrobial resistance in livestock: antimicrobial peptides provide a new solution for a growing challenge. Anim Front. (2018) 8:21–9. 10.1093/af/vfy005 32002215 PMC6951932

[9] Blair JMA Webber MA Baylay AJ Ogbolu DO Piddock LJV. Molecular mechanisms of antibiotic resistance. Nat Rev Microbiol. (2015) 13:42–51. 10.1038/nrmicro3380 25435309

[10] Hu F Wu Q Song S She R Zhao Y Yang Y. Antimicrobial activity and safety evaluation of peptides isolated from the hemoglobin of chickens. BMC Microbiol. (2016) 16:287. 10.1186/s12866-016-0904-3 27919228 PMC5139128

[11] Marshall BM Levy SB. Food animals and antimicrobials: impacts on human health. Clin Microbiol Rev. (2011) 24:718–33. 10.1128/CMR.00002-11 21976606 PMC3194830

[12] Lin J Hunkapiller AA Layton AC Chang YJ Robbins KK. Response of intestinal microbiota to antibiotic growth promoters in chickens. Foodborne Pathog Dis. (2013) 10:331–7. 10.1089/fpd.2012.1348 23461609

[13] Karp BE Tate H Plumblee JR Dessai U Whichard JM Thacker EL. National antimicrobial resistance monitoring system: two decades of advancing public health through integrated surveillance of antimicrobial resistance. Foodborne Pathog Dis. (2017) 14:545–57. 10.1089/fpd.2017.2283 28792800 PMC5650714

[14] Food US and Drug Administration. Development and Approval Process - Animal Drugs and Animal Food Additives. (2020). Available online at: https://www.fda.gov/animal-veterinary/development-approval-process/fact-sheet-veterinary-feed-directive-final-rule-and-next-steps (accessed July 19, 2020).

[15] Chen J Sun R Pan C Sun Y Mai B Li QX. Antibiotics and food safety in aquaculture. J Agric Food Chem. (2020) 68:11908–19. 10.1021/acs.jafc.0c03996 32970417

[16] European Commission. Ban on Antibiotics as Growth Promoters in Animal Feed Enters into Effect. European Commission Press Release Database (2005). Available online at: http://europa.eu/rapid/press-release_IP-05-1687_en.htm (accessed June 13, 2021).

[17] Access Science Editors. U.S. Bans Antibiotics Use for Enhancing Growth in Livestock. New York, NY: McGraw-Hill Education (2017).

[18] Laxminarayan R Matsoso P Pant S Brower C Røttingen JA Klugman K. Access to effective antimicrobials: a worldwide challenge. Lancet (London, England). (2016) 387:168–75. 10.1016/S0140-6736(15)00474-2 26603918

[19] Kasimanickam V Kasimanickam M Kasimanickam R. Antibiotics use in food animal production: escalation of antimicrobial resistance: where are we now in combating AMR? Med Sci (Basel, Switzerland). (2021) 9:14. 10.3390/medsci9010014 33669981 PMC7931007

[20] da Costa JP Cova M Ferreira R Vitorino R. Antimicrobial peptides: an alternative for innovative medicines? Appl Microbiol Biotechnol. (2015) 99:2023–40. 10.1007/s00253-015-6375-x 25586583

[21] Mahlapuu M Håkansson J Ringstad L Björn C. Antimicrobial peptides: an emerging category of therapeutic agents. Front Cell Infect Microbiol. (2016) 6:194. 10.3389/fcimb.2016.00194 28083516 PMC5186781

[22] Pinheiro da Silva F Machado MC. The dual role of cathelicidins in systemic inflammation. Immunol Lett. (2017) 182:57–60. 10.1016/j.imlet.2017.01.004 28082134

[23] Holani R Shah C Haji Q Inglis GD Uwiera RR Cobo ER. Proline-arginine rich (PR-39) cathelicidin: structure, expression and functional implication in intestinal health. Comp Immunol Microbiol Infect Dis. (2016) 49:95–101. 10.1016/j.cimid.2016.10.004 27865272

[24] Veldhuizen EJ Schneider VA Agustiandari H van Dijk A Tjeerdsma-van Bokhoven JL Bikker FJ. Antimicrobial and immunomodulatory activities of PR-39 derived peptides. PLoS ONE. (2014) 9:e95939. 10.1371/journal.pone.0095939 24755622 PMC3995882

[25] Maloy KJ Powrie F. Intestinal homeostasis and its breakdown in inflammatory bowel disease. Nature. (2011) 474:298–306. 10.1038/nature10208 21677746

[26] Gao L Gülcüler GS Golbach L Block H Zarbock A Martin-Villalba A. Endothelial cell-derived CD95 ligand serves as a chemokine in induction of neutrophil slow rolling and adhesion. eLife. (2016) 5:e18542. 10.7554/eLife.18542 27763263 PMC5098908

[27] Yacoub HA Elazzazy AM Abuzinadah OA Al-Hejin AM Mahmoud MM Harakeh SM. Antimicrobial activities of chicken β-defensin (4 and 10) peptides against pathogenic bacteria and fungi. Front Cell Infect Microbiol. (2015) 5:36. 10.3389/fcimb.2015.00036 25941665 PMC4400880

[28] Jiao W Ma Q Lv X Shan A Li Z. Gene expression and tissue distribution of β-defensins in Chinese Min pigs and Landrace pigs. Czech J Anim Sci. (2017) 62:178–83. 10.17221/84/2015-CJAS

[29] Auvynet C Rosenstein Y. Multifunctional host defense peptides: antimicrobial peptides, the small yet big players in innate and adaptive immunity. FEBS J. (2009) 276:6497–508. 10.1111/j.1742-4658.2009.07360.x 19817855

[30] Soman SS Arathy DS Sreekumar E. Discovery of *Anas platyrhynchos* avian beta-defensin 2 (Apl_AvBD2) with antibacterial and chemotactic functions. Mol Immunol. (2009) 46:2029–38. 10.1016/j.molimm.2009.03.003 19362739

[31] Mao X Qi S Yu B Huang Z Chen H Mao Q. Dietary L-arginine supplementation enhances porcine b-defensins gene expression in some tissues of weaned pigs. (2012). 10.1016/j.livsci.2012.05.016

[32] Yi D Li B Hou Y Wang L Zhao D Chen H. Dietary supplementation with an amino acid blend enhances intestinal function in piglets. Amino Acids. (2018) 50:1089–100. 10.1007/s00726-018-2586-7 29770867

[33] Gu C Mao X Chen D Yu B Yang Q. Isoleucine plays an important role for maintaining immune function. Curr Protein Pept Sci. (2019) 20:644–51. 10.2174/1389203720666190305163135 30843485

[34] Song KD Lee WK. Antibacterial activity of recombinant pig intestinal parasite cecropin P4 peptide secreted from Pichia pastoris. Asian Aust J Anim Sci. (2014) 27:278. 10.5713/ajas.2013.13615 25049952 PMC4093210

[35] Wu S Zhang F Huang Z Liu H Xie C Zhang J. Effects of the antimicrobial peptide cecropin AD on performance and intestinal health in weaned piglets challenged with *Escherichia coli*. Peptides. (2012) 35:225–30. 10.1016/j.peptides.2012.03.030 22490448

[36] Mao XF Piao XS Lai CH Li DF Xing JJ Shi BL. Effects of β-glucan obtained from the Chinese herb Astragalus membranaceus and lipopolysaccharide challenge on performance, immunological, adrenal, and somatotropic responses of weanling pigs. J Anim Sci. (2005) 83:2775–82. 10.2527/2005.83122775x 16282615

[37] Amezcua R Friendship RM Dewey CE Gyles C Fairbrother JM. Presentation of postweaning *Escherichia coli* diarrhea in southern Ontario, prevalence of hemolytic *E. coli* serogroups involved, and their antimicrobial resistance patterns. Can J Vet Res. (2002) 66:73. 11989737 PMC226986

[38] Barkema HW Green MJ Bradley AJ Zadoks RN. Invited review: the role of contagious disease in udder health. J Dairy Sci. (2009) 92:4717–29. 10.3168/jds.2009-2347 19762787 PMC2765761

[39] Sahoo BR Maruyama K Edula JR Tougan T Lin Y Lee YH. Mechanistic and structural basis of bioengineered bovine Cathelicidin-5 with optimized therapeutic activity. Sci Rep. (2017) 7:44781. 10.1038/srep44781 28322271 PMC5359555

[40] Olde Riekerink RG Barkema HW Kelton DF Scholl DT. Incidence rate of clinical mastitis on Canadian dairy farms. J Dairy Sci. (2008) 91:1366–77. 10.3168/jds.2007-0757 18349229

[41] Skerlavaj B Gennaro R Bagella L Merluzzi L Risso A Zanetti M. Biological characterization of two novel cathelicidin-derived peptides and identification of structural requirements for their antimicrobial and cell lytic activities. J Biol Chem. (1996) 271:28375–81. 10.1074/jbc.271.45.28375 8910461

[42] Duarte CM Freitas PP Bexiga R. Technological advances in bovine mastitis diagnosis: an overview. J Vet Diagn Invest. (2015) 27:665–72. 10.1177/1040638715603087 26450837

[43] Bowdish DM Davidson DJ Scott MG Hancock RE. Immunomodulatory activities of small host defense peptides. Antimicrob Agents Chemother. (2005) 49:1727–32. 10.1128/AAC.49.5.1727-1732.2005 15855488 PMC1087655

[44] Wieczorek M Jenssen H Kindrachuk J Scott WR Elliott M Hilpert K. Structural studies of a peptide with immune modulating and direct antimicrobial activity. Chem Biol. (2010) 17:970–80. 10.1016/j.chembiol.2010.07.007 20851346

[45] Vulikh K Bassel LL Sergejewich L Kaufman EI Hewson J MacInnes JI. Effect of tracheal antimicrobial peptide on the development of Mannheimia haemolytica pneumonia in cattle. PLoS ONE. (2019) 14:e0225533. 10.1371/journal.pone.0225533 31770402 PMC6879128

[46] Taha-Abdelaziz K Wyer L Berghuis L Bassel LL Clark ME Caswell JL. Regulation of tracheal antimicrobial peptide gene expression in airway epithelial cells of cattle. Vet Res. (2016) 47:44. 10.1186/s13567-016-0329-x 26987959 PMC4797111

[47] Coorens M Schneider VA de Groot AM van Dijk A Meijerink M Wells JM. Cathelicidins inhibit *Escherichia coli*-induced tlr2 and tlr4 activation in a viability-dependent manner. J Immunol. (2017) 199:1418–28. 10.4049/jimmunol.1602164 28710255 PMC5544931

[48] Mukhopadhyaya CS Kumar R Brahc GS. Gallinacin and fowlicidin: two promising antimicrobial peptides in chickens–a review. Vet World Rajkot. (2010) 3:297–300.

[49] Veldhuizen EJ Brouwer EC Schneider VA Fluit AC. Chicken cathelicidins display antimicrobial activity against multiresistant bacteria without inducing strong resistance. PLoS ONE. (2013) 8:e0061964. 10.1371/journal.pone.0061964 23613986 PMC3632573

[50] Goitsuka R Chen CL Benyon L Asano Y Kitamura D Cooper MD. Chicken cathelicidin-B1, an antimicrobial guardian at the mucosal M cell gateway. Proc Natl Acad Sci USA. (2007) 104:15063–8. 10.1073/pnas.0707037104 17827276 PMC1986613

[51] Bommineni YR Pham GH Sunkara LT Achanta M Zhang G. Immune regulatory activities of fowlicidin-1, a cathelicidin host defense peptide. Mol Immunol. (2014) 59:55–63. 10.1016/j.molimm.2014.01.004 24491488

[52] Sunkara LT Zeng X Curtis AR Zhang G. Cyclic AMP synergizes with butyrate in promoting β-defensin 9 expression in chickens. Mol Immunol. (2014) 57:171–80. 10.1016/j.molimm.2013.09.003 24141182

[53] van Dijk A Veldhuizen EJ Haagsman HP. Avian defensins. Vet Immunol Immunopathol. (2008) 124:1–18. 10.1016/j.vetimm.2007.12.006 18313763 PMC7112556

[54] Cuperus T Coorens M van Dijk A Haagsman HP. Avian host defense peptides. Dev Comp Immunol. (2013) 41:352–69. 10.1016/j.dci.2013.04.019 23644014

[55] Lim W Jeong W Kim J Yoshimura Y Bazer FW Han JY. Expression and regulation of beta-defensin 11 in the oviduct in response to estrogen and in ovarian tumors of chickens. Mol Cell Endocrinol. (2013) 366:1–8. 10.1016/j.mce.2012.10.031 23159989

[56] Whenham N Lu TC Maidin MB Wilson PW Bain MM Stevenson ML. Ovodefensins, an oviduct-specific antimicrobial gene family, have evolved in birds and reptiles to protect the egg by both sequence and intra-six-cysteine sequence motif spacing. Biol Reprod. (2015) 92:154–1. 10.1095/biolreprod.114.126839 25972010

[57] Hervé V Meudal H Labas V Réhault-Godbert S Gautron J Berges M. Three-dimensional NMR structure of hen egg gallin (chicken ovodefensin) reveals a new variation of the β-defensin fold. J Biol Chem. (2014) 289:7211–20. 10.1074/jbc.M113.507046 24443564 PMC3945380

[58] Gong D Wilson PW Bain MM McDade K Kalina J Hervé-Grépinet V. Gallin; an antimicrobial peptide member of a new avian defensin family, the ovodefensins, has been subject to recent gene duplication. BMC Immunol. (2010) 11:12. 10.1186/1471-2172-11-12 20226050 PMC2846878

[59] Katzenback BA. Antimicrobial peptides as mediators of innate immunity in teleosts. Biology. (2015) 4:607–39. 10.3390/biology4040607 26426065 PMC4690011

[60] Niu SF Jin Y Xu X Qiao Y Wu Y Mao Y. Characterization of a novel piscidin-like antimicrobial peptide from Pseudosciaena crocea and its immune response to Cryptocaryon irritans. Fish Shellfish Immunol. (2013) 35:513–24. 10.1016/j.fsi.2013.05.007 23727503

[61] Wang YD Kung CW Chen JY. Antiviral activity by fish antimicrobial peptides of epinecidin-1 and hepcidin 1-5 against nervous necrosis virus in medaka. Peptides. (2010) 31:1026. 10.1016/j.peptides.2010.02.025 20214942

[62] Pinzon-Arango PA Nagarajan R Camesano TA. Interactions of antimicrobial peptide chrysophsin-3 with *Bacillus anthracis* in sporulated, germinated, and vegetative states. J Phys Chem. (2013) 117B:6364–72. 10.1021/jp400489u 23631815

[63] Mulero I Noga EJ Meseguer J Garcia-Ayala A Mulero V. The antimicrobial peptides piscidins are stored in the granules of professional phagocytic granulocytes of fish and are delivered to the bacteria-containing phagosome upon phagocytosis. Dev Comp Immunol. (2008) 32:1531–8. 10.1016/j.dci.2008.05.015 18582499

[64] Peng KC Lee SH Hour A-L Pan CY Lee LH Chen J. Five different piscidins from Nile tilapia, *Oreochromis niloticus*: analysis of their expressions and biological functions. PLoS ONE. (2012) 7:e50263. 10.1371/journal.pone.0050263 23226256 PMC3511469

[65] Huang HN Rajanbabu V Pan CY Chan YL Wu CJ Chen JY. Use of the antimicrobial peptide Epinecidin-1 to protect against MRSA infection in mice with skin injuries. Biomaterials. (2013) 34:10319–27. 10.1016/j.biomaterials.2013.09.037 24075409

[66] Wang G Li J Zou P Xie H Huang B Nie P. Expression pattern, promoter activity and bactericidal property of beta-defensin from the mandarin fish *Siniperca chuatsi*. Fish Shellfish Immunol. (2012) 33:522–31. 10.1016/j.fsi.2012.06.003 22705342

[67] Guo M Wei J Huang X Huang Y Qin Q. Antiviral effects of beta-defensin derived from orange-spotted grouper (*Epinephelus coioides*). Fish Shellfish Immunol. (2012) 32:828–38. 10.1016/j.fsi.2012.02.005 22343108

[68] Ruangsri J Kitani Y Kiron V Lokesh J Brinchmann MF Karlsen BO. A novel beta-defensin antimicrobial peptide in Atlantic cod with stimulatory effect on phagocytic activity. PLoS ONE. (2013) 8:e62302. 10.1371/journal.pone.0062302 23638029 PMC3636224

[69] Broekman DC Guethmundsson GH Maier VH. Differential regulation of cathelicidin in salmon and cod. Fish Shellfish Immunol. (2013) 35:532–8. 10.1016/j.fsi.2013.05.005 23727282

[70] Mao MG Jiang JL Peralvarez-Marin A Wang KJ Lei JL. Characterization of the Mx and hepcidin genes in *Epinephelus akaara* asymptomatic carriers of the nervous necrosis virus. Aquaculture. (2013) 408:175–83. 10.1016/j.aquaculture.2013.05.039

[71] Neves JV Caldas C Vieira I Ramos MF Rodrigues PN. Multiple hepcidins in a teleost fish, *Dicentrarchus labrax*: different hepcidins for different roles. J Immunol. (2015) 195:2696–709. 10.4049/jimmunol.1501153 26268656

[72] Pietrangelo A Trautwein C. Mechanisms of disease: the role of hepcidin in iron homeostasis-implications for hemochromatosis and other disorders. Nat Clin Pract Gastroenterol Hepatol. (2004) 1:39–45. 10.1038/ncpgasthep0019 16265043

[73] Fraenkel PG Traver D Donovan A Zahrieh D Zon LI. Ferroportin1 is required for normal iron cycling in zebrafish. J Clin Investig. (2005) 115:1532–41. 10.1172/JCI23780 15902304 PMC1089797

[74] Rodrigues PNS Vazquez-Dorado S Neves JV Wilson JM. Dual function of fish hepcidin: response to experimental iron overload and bacterial infection in sea bass (*Dicentrarchus labrax*). Dev Comp Immunol. (2006) 30:1156–67. 10.1016/j.dci.2006.02.005 16616368

[75] Patrzykat A Zhang L Mendoza V Iwama GK Hancock RE. Synergy of histone-derived peptides of coho salmon with lysozyme and flounder pleurocidin. Antimicrob Agents Chemother. (2001) 45:1337–42. 10.1128/AAC.45.5.1337-1342.2001 11302792 PMC90470

[76] Bechinger B Gorr SU. Antimicrobial peptides: mechanisms of action and resistance. J Dental Res. (2017) 96:254–60. 10.1177/0022034516679973 27872334 PMC5298395

[77] Brzoza P Godlewska U Borek A Morytko A Zegar A Kwiecinska P. Redox active antimicrobial peptides in controlling growth of microorganisms at body barriers. Antioxidants (Basel, Switzerland). (2021) 10:446. 10.3390/antiox10030446 33805777 PMC7998263

[78] Bauer ME Shafer WM. On the *in vivo* significance of bacterial resistance to antimicrobial peptides. Biochimica et biophysica acta. (2015) 1848:3101–11. 10.1016/j.bbamem.2015.02.012 25701234 PMC4540701

[79] Yeaman MR Yount NY. Mechanisms of antimicrobial peptide action and resistance. Pharmacol Rev. (2003) 55:27–55. 10.1124/pr.55.1.2 12615953

[80] Hancock RE. Cationic peptides: effectors in innate immunity and novel antimicrobials. Lancet Infect Dis. (2001) 1:156–64. 10.1016/S1473-3099(01)00092-5 11871492

[81] Abdi M Mirkalantari S Amirmozafari N. Bacterial resistance to antimicrobial peptides. J Peptide Sci. (2019) 25:e3210. 10.1002/psc.3210 31637796

[82] Cole JN Nizet V. Bacterial evasion of host antimicrobial peptide defenses. Microbiol Spectr. (2016) 4, 413–43. 10.1128/microbiolspec.VMBF-0006-2015 26999396 PMC4804471

[83] Joo HS Fu CI Otto M. Bacterial strategies of resistance to antimicrobial peptides. Philos Trans R Soc London B Biol Sci. (2016) 371:20150292. 10.1098/rstb.2015.0292 27160595 PMC4874390

[84] Mansour SC Pena OM Hancock RE. Host defense peptides: front-line immunomodulators. Trends Immunol. (2014) 35:443–50. 10.1016/j.it.2014.07.004 25113635

[85] Wu J Ma N Johnston LJ Ma X. Dietary nutrients mediate intestinal host defense peptide expression. Adv Nutr (Bethesda, Md.). (2020) 11:92–102. 10.1093/advances/nmz057 31204774 PMC7442325

[86] Jaeger SU Schroeder BO Meyer-Hoffert U Courth L Fehr SN Gersemann M. Cell-mediated reduction of human β-defensin 1: a major role for mucosal thioredoxin. Mucosal Immunol. (2013) 6:1179–90. 10.1038/mi.2013.17 23571504 PMC3806438

